# Identifying gene expression signatures of oncolytic virus response in patient-derived pancreatic ductal adenocarcinoma organoids

**DOI:** 10.1016/j.omton.2025.201064

**Published:** 2025-09-23

**Authors:** Marco Huberts, Elham Aida Farshadi, Farzana Mohammad, Jie Ju, Andrew Stubbs, Ron A.M. Fouchier, Bernadette G. van den Hoogen

**Affiliations:** 1 Viroscience Department, Erasmus Medical Centrum, Rotterdam, the Netherlands; 2 Department of Pulmonary Medicine, Erasmus University Medical Center, Rotterdam, the Netherlands; 3 Department of Pathology and Clinical Bioinformatics, Erasmus Medical Centrum, Rotterdam, the Netherlands

**Keywords:** MT: Regular Issue, pancreatic ductal adenocarcinoma, viro-immunotherapy, oncolytic viruses, patient-derived organoids, biomarkers, personalized medicine

## Abstract

Pancreatic ductal adenocarcinoma is among the deadliest cancers, with a poor prognosis. Viro-immunotherapy using oncolytic viruses represents a promising treatment. However, pancreatic ductal adenocarcinoma from different patients responds variably to these therapies, highlighting the need for predictive biomarkers. This study aimed to identify gene expression profiles that predict responses to oncolytic viruses. Patient-derived organoids (PDOs) from ten pancreatic ductal adenocarcinoma patients were evaluated for sensitivity to Newcastle disease virus (NDV), reovirus (RV), measles virus, and a gorilla-derived adenovirus. The sensitivity data revealed heterogeneous responses, with nine of ten PDOs being sensitive to at least one virus. The sensitivity of PDOs was correlated with their baseline transcriptome, resulting in gene expression profiles associated with sensitivity to each oncolytic virus. Gene Ontology analysis of the gene expression profiles revealed that intracellular aberrations, particularly those involved in embryonic development, were primary determinants of sensitivity. Additionally, for some oncolytic viruses (OVs), the gene expression profiles linked to sensitivity were associated with genes regulating cell cycle, metabolism, and cell proliferation. Screening tumors for these gene profiles may aid in selecting effective viral treatments for pancreatic ductal adenocarcinoma, providing the stepping stone toward personalized viro-immunotherapy.

## Graphical abstract

**Figure:**
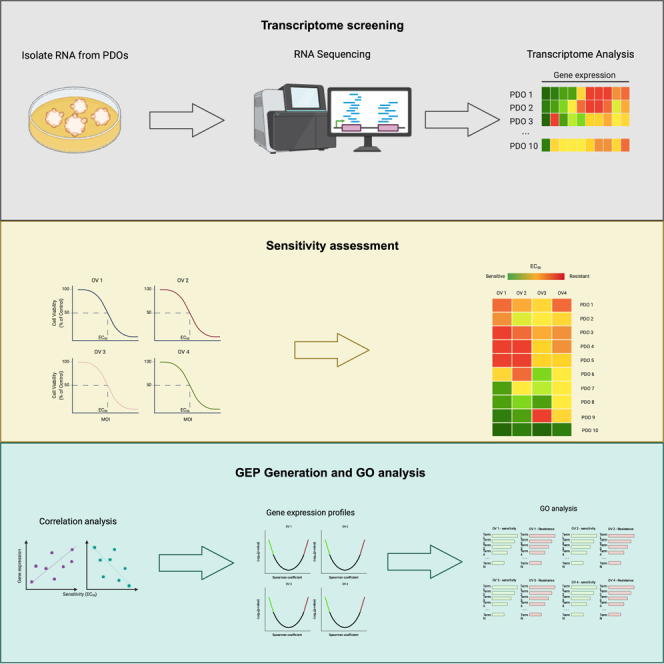

## Teaser

Correlating transcriptomes of patient-derived pancreatic cancer organoids with their sensitivity to oncolytic viruses revealed predictive gene expression profiles for treatment response. These profiles contained genes associated with embryonic development, proliferation, and metabolism, supporting transcriptome-based stratification for personalized oncolytic virus therapy.

## Introduction

Pancreatic cancer is a significant contributor to cancer-associated mortality, accounting for approximately 4.5% of all cancer-related deaths worldwide.^1^ The most prevalent pancreatic cancer, pancreatic ductal adenocarcinoma (PDAC), is associated with poor prognosis with an average 5-year survival rate of less than 10%.^2^ Despite the use of currently available treatment modalities such as gemcitabine and FOLFIRINOX, the prognosis remains poor, emphasizing the need for novel therapeutic strategies. One promising treatment for PDAC is viro-immunotherapy, which uses viruses that selectively kill tumor cells while concurrently triggering an anti-tumor immune response. These viruses are commonly referred to as oncolytic viruses (OVs). Several viruses, including Newcastle disease virus (NDV), measles virus, reovirus (RV), and adenoviruses (AdV) have shown to be effective as OV in a range of tumor models.^3^^,^^4^^,^^5^^,^^6^

As every patient responds differently to OV therapies,^7^ identifying biomarkers that can predict patient responses to these therapies is critical for the effective treatment of PDAC patients. Tumor patient-derived organoids (PDOs), 3D structures of patient-derived cancer cells,^8^ are a suitable model to identify predictive biomarkers as PDOs accurately represent the genetic background and closely resemble the phenotype of the original tumors, even after prolonged culturing.^9^^,^^10^^,^^11^^,^^12^ Consequently, PDOs are increasingly used as predictive models to evaluate the effect of anticancer drugs, including OV therapies, with the aim to develop personalized therapies.^9^^,^^13^ Although PDOs are near-patient models, they remain engineered *in vitro* systems. PDOs complement, rather than replace, scalable cell-based platforms that enable high-throughput discovery. For OVs, personalized therapies will require biomarkers with predictive value for sensitivity, and gene expression profiles (GEPs) are among such promising markers. Here, we nominate baseline GEPs associated with sensitivity to four OVs: a non-virulent strain of NDV, the Edmonston vaccine strain of measles virus (MeV-Edm), a RV strain that can infect cells independently of JAM-A cell surface expression (RV Jin-3) and a gorilla-derived attenuated human AdV strain (Goravir).^3^^,^^5^^,^^14^^,^^15^

## Results

### Establishing and characterizing ten PDAC PDOs

**Figure 1:**
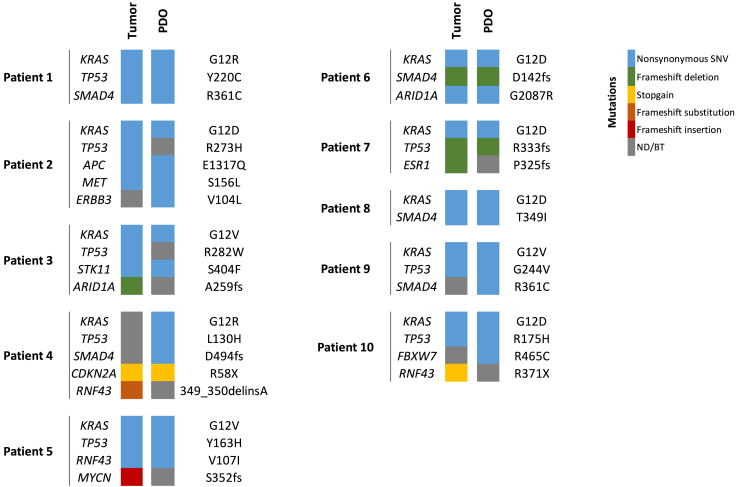
Mutational landscape of PDAC primary tissue and its derived organoids Mutations in 56 cancer-associated signature genes from primary PDAC tissue and corresponding patient-derived organoids (PDOs) were analyzed using next-generation sequencing. The variant allele frequency (VAF) cut-off was set at 8% resulting in the identification of 13 genes with various mutations. The detected mutation types are indicated with colored boxes: nonsynonymous single nucleotide variants (SNV) were labeled blue, frameshift deletions were labeled green, stop gain mutations were labeled yellow, frameshift substitutions were labeled orange and frameshift insertions were labeled red. Mutations that were not detected (ND) or were below threshold (BT) were labeled gray. If multiple mutations were detected in the same gene, only one is shown.

A library of ten PDOs was generated from patients with resectable or borderline resectable PDAC, where three patients received seven to eight cycles of neoadjuvant FOLFIRINOX, a combination chemotherapy consisting of 5-fluorouracil, irinotecan, and oxaliplatin,^16^ and seven patients did not receive any neoadjuvant therapy. Moreover, patient tumors were resected at various stages of disease, as classified by the tumor, node, metastasis (TNM) system, ranging from early-stage IA to more advanced stage III. Most patients had moderately differentiated tumors, while one patient had a tumor with poor-to-moderate differentiation, and another had a poorly differentiated tumor (Table 1). Overall, a heterogeneous library of PDAC PDOs was established. To determine whether these PDOs genetically recapitulated the original tumor material, both primary tumors and related PDOs were assessed for the presence of signature PDAC mutations. To this end, 58 cancer-associated genes were screened using next-generation sequencing (Table S1). Genes with a VAF greater than 8% were included, leading to the identification of 13 genes harboring various mutations across the ten tumors and the related PDOs.^17^ This assessment revealed that almost all primary tumor material and related PDOs, with the exception of those of patient 4, contained a nonsynonymous single nucleotide variant in the *KRAS* gene, which is one of the key PDAC driver mutations (Figure 1). In patient 4, the *KRAS* mutation was found in the PDOs but not in the primary tumor material. However, both primary material and PDO from patient 4 contained a mutation in *CDKN2A*, another well-known driver mutation of PDAC, confirming the PDAC origin of this tumor material. Overall, PDOs closely resembled the original patient tumors, although variability in key driver mutations was observed between patients.

**Table 1:** Clinical characteristics of PDAC patients

	Treatment-naive							FFX-treated		
PID	1	2	3	4	6	8	9	5	7	10
Sex/age	F/68	M/78	F/75	M/70	M/79	F/69	M/69	M/74	M/69	F/55
TNM category at diagnosis^a^	cT2N0M0	cT1N0M0	cT1N1M0	cT2N0M0	cT2N0M0	cT1N0M0	cT2N1M0	cT2N1M0	cT2N1M0	cT2N2M0
Stage^a^	IB	IA	IIB	IB	IB	IA	IIB	llB	IIB	III
Neoadjuvant therapy	none	none	none	none	none	none	none	8× FFX	8× FFX	7× FFX
Biochemical response^b^	N/A	N/A	N/A	N/A	N/A	N/A	N/A	−279	−124	−1
Radiologic response^c^	N/A	N/A	N/A	N/A	N/A	N/A	N/A	SD	PR	SD
Histopathologic response^d^	N/A	N/A	N/A	N/A	N/A	N/A	N/A	partial	partial	partial
TNM category at resection^a^	pT3N0	pT2N0	pT1cN1	pT3N2	pT3N3	pT2N1	pT2N1	ypT2N1	ypT2N2	ypT2N2
Tumor differentiation	moderate	poor-moderate	moderate	moderate	moderate	moderate	moderate	moderate	poor	moderate
Resection margin^e^	R0	R1	R1	R1	R2	R1	R1	R0	R0	R1
Adjuvant therapy	11× FFX	2× FFX	none	4× FFX	none	14× GEM	none	none	none	none
Recurrence	no	no	yes	yes	yes	yes	yes	yes	yes	yes
RFS (months)	57	24	2	6	6	5	12	8	2	2
OS (months)	57	24	10	20	6	11	21	15	10	11

DC, dendritic cells; FFX, FOLFIRINOX; GEM, gemcitabine; OS, overall survival; PR, partial response; RFS, recurrence-free survival; SD, stable disease.

Overall survival (OS) -> by patient which are alive -> to calculate the OS, the last date of registration of the patient at the hospital is used.

a According to AJCC 8th edition staging system.

b The change in CA19-9 levels between before and after chemotherapy.

c According to the RECIST criteria version 1.1.

d Partial regression = >5% rest tumor.

e According to the definition of the UK Royal College of Pathologists

**Figure 2:**
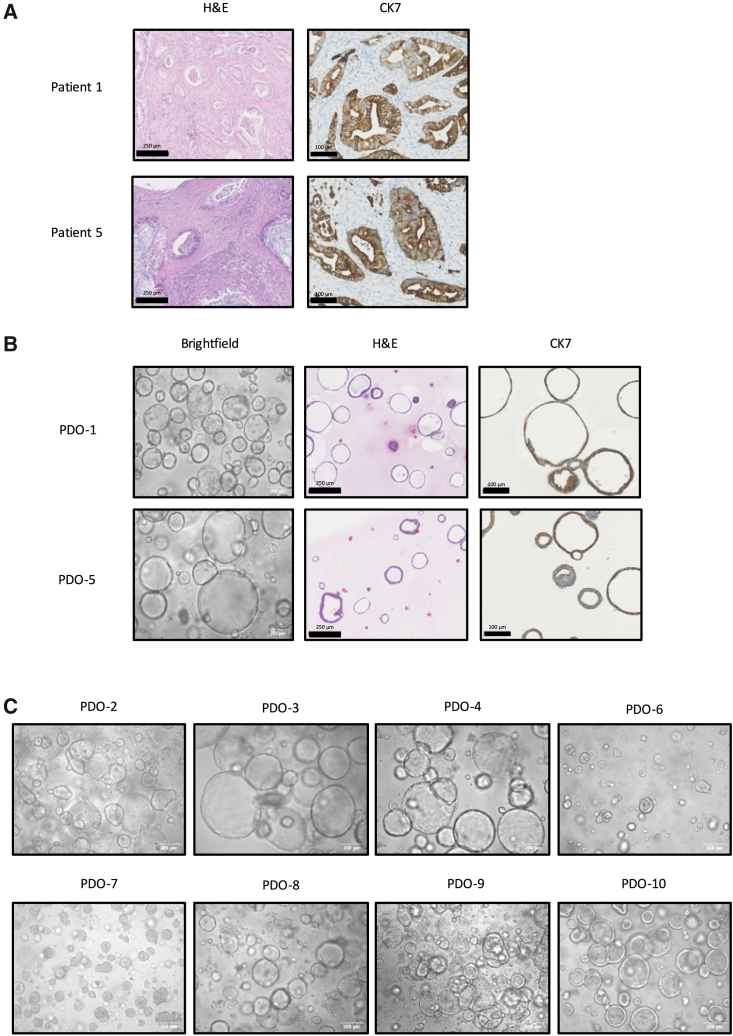
Phenotypical features of patient-derived PDAC tissue and its derived PDOs (A) Representative images of H&E staining (left) and immunohistochemical CK7 staining (right) of sectioned primary PDAC tissue from patient 1 and 5. Scale bars are 250 μm (left) and 100 μm (right). (B) Representative bright field images (left), H&E staining (middle), and immunohistochemical CK7 staining (right) of PDO-1 and PDO-5. Scale bars are 200 μm (left), 250 μm (middle), and 100 μm (right). (C) Representative bright field images of all PDOs except PDO-1 and PDO-5. Most PDOs had a cystic phenotype, while only PDO-6 and PDO-7 had a dense phenotype. Scale bars are 200 μm.

To assess phenotypical characteristics, histological and immunohistochemical analyses were performed on original tumor material and the related PDO from one treatment-naive (patient 1) and one FOLFIRINOX-treated patient (patient 5), as representatives of these contrasting treatments. H&E staining of the tumor material revealed distinct morphological differences between the two patients, with patient 5 exhibiting a higher density of nuclei compared to patient 1 (Figure 2A). Immunohistochemistry (IHC) staining for keratin-7 (CK7), a well-established PDAC marker, revealed its expression in tumor material from both patients. In both the primary tumor and in the PDOs, high expression of CK7 was observed supporting their PDAC origin, without profound differences in CK7 expression between the treated and non-treated patients (Figure 2B). In addition, in the PDOs, the H&E staining also did not reveal major differences that could be linked to FOLFIRINOX treatment. Bright-field microscopy of the remaining PDOs revealed that PDO-6 and PDO-7 exhibited a dense phenotype, characterized by the absence of lumen in the cellular aggregates. The other PDOs displayed a cystic phenotype, characterized by the presence of a liquid-filled lumen (Figure 2C).

### Sensitivity of PDOs to a panel of OVs

**Figure 3:**
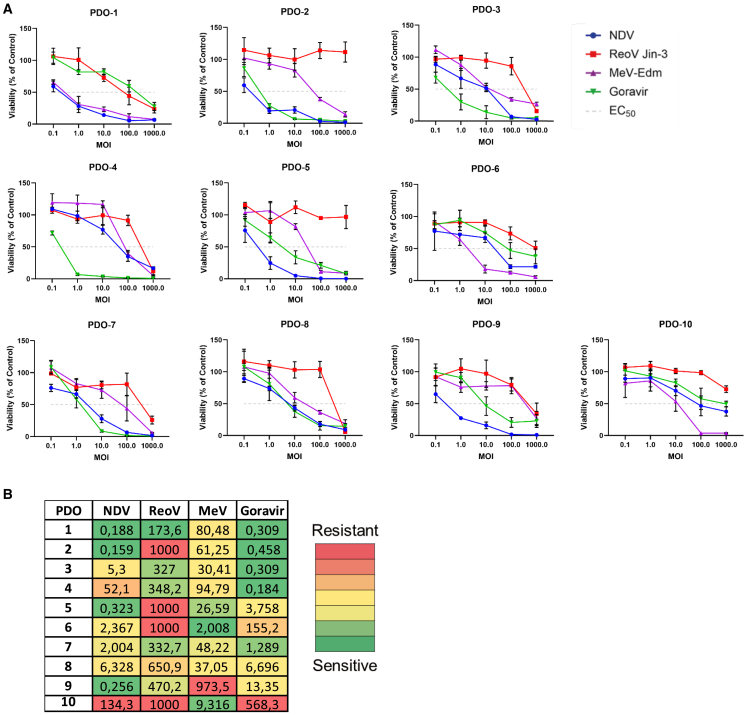
Sensitivity of PDAC PDOs to NDV, MeV-Edm, RV Jin-3, and Goravir (A) Viability of ten PDOs inoculated with NDV, MeV-Edm, reovirus, or Goravir at the indicated multiplicities of infection (MOIs). The dotted line indicates 50% viability, representing the EC_50_ value threshold. Data are presented as the percentage of surviving cells compared to mock-treated cells that were considered 100% viable. Experiments were conducted in triplicate and mean and standard deviation are depicted. (B) The EC_50_ values, represented as MOIs for the four OVs in each PDO, color-coded from green to red based on their respective EC_50_ values per OV.

To determine the sensitivity to the selected OVs, PDOs were inoculated with NDV, RV Jin-3, MeV-Edm, and Goravir at multiplicities of infection (MOIs) ranging from 0.1 to 1,000. Upon assessment of viability at five days post inoculation, the PDOs revealed variability in sensitivity to the various OVs (Figure 3A). Based on these data, the half maximal effective concentration (EC_50_) value was determined for each OV in each PDO. Each EC_50_ value was visually represented using a red-yellow-green gradient scale: green indicating the lowest EC_50_ values (indicating higher sensitivity), yellow the intermediate EC_50_ values (moderate sensitivity), and red the highest EC_50_ values (lower sensitivity). This resulted in a heatmap displaying the heterogenic sensitivity of PDOs to the variety of OVs (Figure 3B). The color gradient, ranging from green to red, corresponded to the EC50 values observed for each OV. Notably, in four PDOs treated with RV Jin-3, even the highest MOI tested, an MOI of 1,000, failed to induce 50% cell death. These cases were therefore annotated as “>1,000” in the heatmap. The heatmap also suggests that pre-treatment with FOLFIRINOX possibly did not impact PDO sensitivity to OV therapies, as shown by PDO-5 being sensitive to NDV while PDO-10 was not, while both being pre-treated with FOLFIRINOX.

### Identifying GEPs correlated to OV sensitivity and resistance

**Figure 4:**
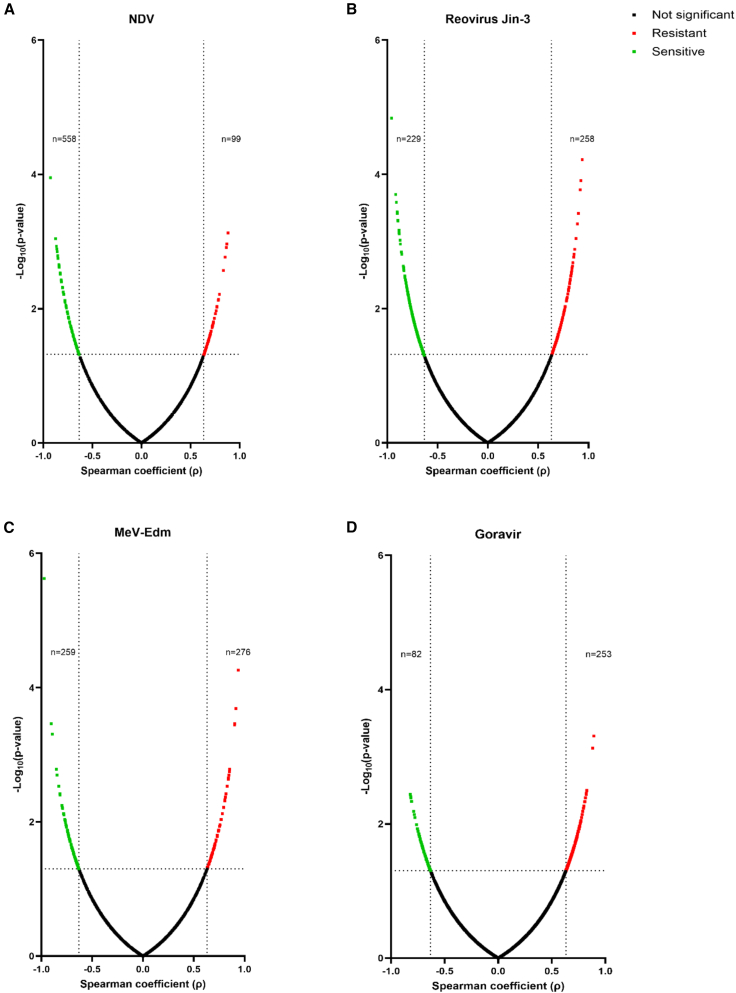
Identification of GEPs associated with sensitivity of PDOs to OVs Volcano plots displaying the Spearman’s correlation coefficients (ρ) of genes that were significantly correlated with sensitivity or resistance to (A) NDV, (B) RV Jin-3, (C) MeV-Edm, and (D) Goravir. Correlations are based on gene expression per PDO and the accompanying EC_50_ values. The *x* axis shows the Spearman’s correlation coefficient (ρ), while the *y* axis represents the negative logarithm of the *p* value (−log_10_ [*p* value]). The cut-off for statistical significance was set at *p* = 0.05. Genes with non-significant correlations are highlighted in black, while genes with significant positive correlations, associated with resistance to OV treatment, are highlighted in red. Contrarily, genes with significant negative correlations, associated with sensitivity to OV treatment, are highlighted in green.

To identify GEPs associated with sensitivity to each OV, transcriptomes of uninfected PDOs were generated and correlated with the EC_50_ values of the OVs per PDO, resulting in Spearman’s correlation coefficients (rho; ρ) and the associated *p* values (Figure 4). For NDV, the GEP associated with sensitivity contained profoundly more genes compared to the GEP associated with resistance (Figure 4A). For both RV Jin-3 and MeV-Edm, the GEPs associated with sensitivity and resistance contained a comparable number of genes (Figures 4B and 4C). For Goravir, the GEP associated with resistance included more genes than the GEP associated with sensitivity (Figure 4D). The genes that made up the GEPs associated with sensitivity and resistance to each OV are summarized in Table S2. Altogether, this analysis identified GEPs that could potentially be used for further development of predictive markers for sensitivity to each OV.

### Identification of biological processes linked to GEPs associated with sensitivity and resistance

**Figure 5:**
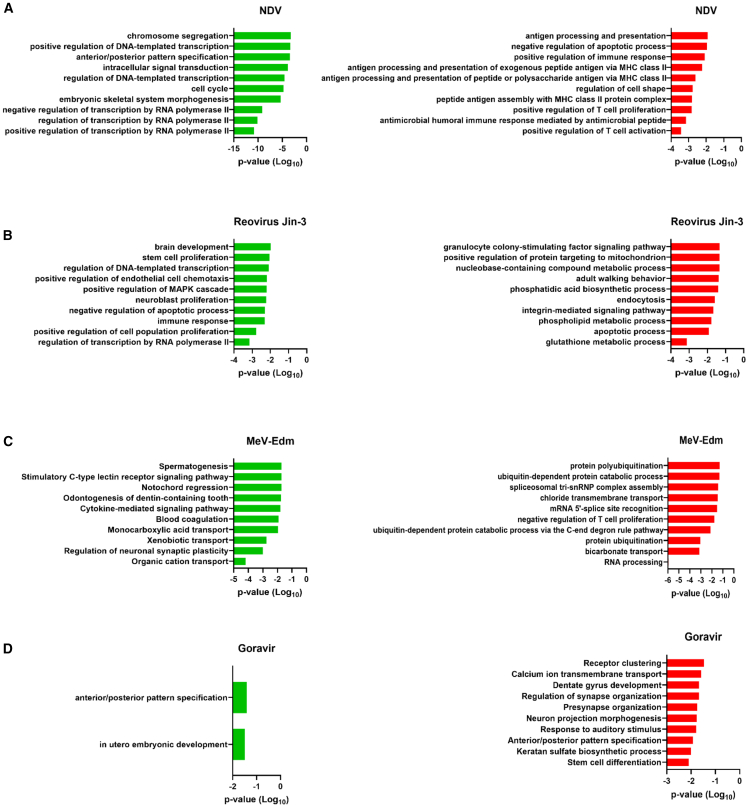
Gene Ontology analysis of genes related to sensitivity or resistance to OVs (A–C) For genes associated with sensitivity and resistance to (A) NDV, (B) RV Jin-3, and (C) MeV-Edm, the ten most significant biological processes were selected when more than ten were identified. (D) For Goravir, only two significant biological processes were identified for sensitivity due to the limited number of significant genes in the GEP, while the ten most significant processes were selected for resistance. The threshold for statistical significance was set at *p* = 0.05.

To identify the biological processes linked to the identified GEPs, the GEPs were analyzed using the Gene Ontology database. This revealed significant associations between several biological processes and sensitivity to each OV (Figure 5). In general, GEPs associated with sensitivity were frequently linked to biological processes such as cell growth, embryonic development, and cell cycle regulation suggesting these biological processes may sensitize cells to OV therapy. In contrast, resistance-associated GEPs were frequently linked with immune responses and cell survival mechanisms (Figure 5). The main category of biological processes linked with sensitivity to all four OVs was embryonic development, with Gene Ontology terms such as “anterior/posterior pattern specification,” “stem cell proliferation,” “brain development,” “neuroblast proliferation,” and “embryonic skeletal system morphogenesis.”

For each OV various biological processes were identified related to critical OV-related pathways. For example, genes associated with NDV sensitivity were linked to the Gene Ontology terms “cell cycle,” “positive regulation DNA-templated transcription,” and “positive regulation of transcription by RNA polymerase II,” suggesting that cells in a highly proliferative state are more sensitive to NDV. Resistance to NDV was characterized by Gene Ontology terms mainly associated with immune responses, such as “positive regulation of immune response,” “peptide antigen assembly with MHC class II protein complex,” and “positive regulation of T cell activation,” implying that strong immune responses reduce sensitivity to NDV (Figure 5A). Sensitivity to RV Jin-3 was associated with the Gene Ontology terms “positive regulation of MAPK cascade,” “positive regulation of cell population proliferation,” and “stem cell proliferation,” indicating that a highly proliferative state is also associated with the sensitivity of PDAC PDOs to RV Jin-3. Resistance to RV Jin-3 was associated with GO terms related to metabolic processes such as “glutathione metabolic process,” “phospholipid metabolic process,” and “nucleobase-containing compound metabolic process,” highlighting that a high metabolic state might reduce sensitivity to RV Jin-3 (Figure 5B).

The GEP linked to sensitivity to MeV-Edm was associated with “regulation of neuronal synaptic plasticity,” suggesting that cells with nervous system-related properties may be more susceptible to this OV. In addition, upregulation of genes involved in biological processes such as “monocarboxylic acid transport,” “xenobiotic transport,” and “organic cation transport,” indicates that cells in a specific metabolic state are sensitive to MeV-Edm. Conversely, GO terms such as “protein polyubiquitination,” “protein ubiquitination,” “ubiquitin-dependent protein catabolic process,” and “ubiquitin-dependent protein catabolic process via the C-end degron rule pathway,” suggests that a highly catabolic state is linked to resistance to MeV-Edm (Figure 5C). Resistance to Goravir was associated with nervous system-related processes, such as “regulation of synapse organization,” “presynapse organization,” and “neuron projection morphogenesis,” highlighting Goravir’s lack of neurotropism. Additionally, the Gene Ontology term “stem cell differentiation” was linked with resistance to Goravir, suggesting that cancer cells lacking stem cell characteristics are less sensitive to OV therapies involving Goravir (Figure 5D). Altogether, these findings highlight the biological relevance of the genes within the identified GEPs.

## Discussion

OVs have often been shown to be effective only in a subset of PDAC patients.^4^^,^^15^^,^^18^^,^^19^^,^^20^ While clinical trials make it challenging to attribute resistance to OV therapy solely to the genetic background of tumors, genetic factors are known to play a crucial role in therapy sensitivity.^21^ Identifying predictive markers, such as GEPs, is essential to determining the most effective OV therapy for each PDAC patient.

Three-dimensional near-patient models, such as PDOs, have shown to accurately recapitulate the characteristics of the original tumor tissue, making them a valuable model for generating GEPs with predictive value for treatment effectivity.^22^ Other studies have similarly utilized near-patient models to generate GEPs associated with sensitivity to OV therapy, further supporting their relevance in personalized treatment strategies.^23^^,^^24^ Despite their accurate representation of patient tumor genetic backgrounds, PDOs remain synthetic *in vitro* models grown in defined matrices and lacking the full tumor microenvironment. Their handling requirements and sensitivity to culture conditions make them less-suited for the throughput achievable in large 2D cell line collections. We therefore view cell line screens as complementary discovery platforms, with PDOs used to refine and orthogonally test biomarker hypotheses before subsequent *in vivo* and clinical validation.

However, GEPs associated with sensitivity of PDAC PDOs to NDV, RV Jin-3, MeV-Edm, or Goravir have not yet been generated. Here, baseline transcriptomes of ten PDAC PDOs were correlated with OV EC_50_ values to nominate GEPs associated with sensitivity to each virus. As expected, PDAC PDOs exhibited heterogeneous responses to the four OVs, which is in line with observations in other studies using different OVs.^23^^,^^24^^,^^25^ Notably, 9 out of 10 PDOs showed sensitivity to at least one of the four tested OVs, indicating that often one OV is a suitable treatment option for the patient from which the PDO was established. Furthermore, GEPs were generated that were associated with sensitivity to each OV, whereby embryonic development was linked with sensitivity to all four OVs. This aligns with findings from another study where embryonic development-related processes in PDAC monolayers were linked to sensitivity to RV and MeV variants.^25^ Moreover, it is known that embryonic development processes are driven by undifferentiated cells that exhibit stem cell-like characteristics, such as self-renewal and differentiation potential.^26^ Several other studies have shown that cells possessing these characteristics are more susceptible to OV therapy.^27^^,^^28^^,^^29^ Gene Ontology database analysis identified only a limited number of significant biological processes linked with sensitivity to Goravir, most likely due to the small number of significant genes (82) in the GEP associated with sensitivity to Goravir. Therefore, further in-depth analysis of each gene in this GEP is needed to further explore their role in relevant biological processes.

Various biological processes associated with sensitivity varied between OVs. For example, sensitivity to NDV and RV Jin-3 was associated with a highly proliferative state, which has also been observed in other studies evaluating the sensitivity of glioblastoma and pancreatic cancer models to OVs.^24^^,^^25^ Specifically for RV Jin-3, sensitivity was associated with the Gene Ontology term “positive regulation of MAPK cascade,” a pathway downstream of RAS that is frequently overexpressed in malignancies sensitive to RVs and plays an important role in cell proliferation and survival.^30^^,^^31^

Upregulation of nervous system-related processes was associated with resistance to Goravir, indicating its lack of neurotropism. Several studies have shown that modification of the adenoviral fiber knob with an RGD motif enhanced neural tissue tropism and improved viral efficacy in glioma models.^32^^,^^33^^,^^34^ Incorporating a similar motif in Goravir could potentially help overcome resistance to cells with upregulated nervous system-related processes. Although different metabolic processes were associated with sensitivity to MeV-Edm and resistance to RV Jin-3, the underlying reason for this discrepancy remains unclear. Altogether, these identified biological processes emphasize the relevance of the genes within the identified GEPs in relation to OV therapy and highlight the potential of these GEPs to predict responsiveness to OV treatment.

Several studies suggested that the primary determinant for sensitivity to OVs is not the expression of virus receptors on cancer cells, but rather intracellular abnormalities.^24^^,^^25^ Our data support this conclusion, as limited biological processes from the Gene Ontology database linked to OV sensitivity were related to cell surface receptor expression. However, one notable exception is the Gene Ontology term “receptor clustering,” which was linked with resistance to Goravir, indicating that altered cell surface protein-related processes could potentially hinder Goravir-induced cell death. Aside from this exception, our findings support the idea that intracellular abnormalities, rather than the expression of cell surface receptors, are likely the key factors in determining sensitivity to OV therapy.

PDOs are known to accurately capitulate the genetic profile of the original tumor tissue.^35^ Sequencing of PDOs and original tumors revealed that most PDOs and related patient samples shared the same key driver mutations, confirming their genetic similarity and reinforcing the PDOs’ predictive value. However, the original tumor from patient 4 lacked *KRAS*, *TP53*, and *SMAD4* mutations, which were present in the PDO. The discrepancy can likely be attributed to the low tumor cellularity of the original tumor material, as observed by the pathologist. The tumor material was predominantly composed of stroma, making it challenging to detect mutations representative of the entire tumor. This suggests that expansion of cells derived from low cellularity tumors through the generation of PDOs can improve the detection of key driver mutations that may be missed in stromal-rich tumor samples.

To select relevant key driver mutations in both patient tumor samples and PDOs, a VAF threshold of 8% was set. While no universal VAF threshold exists for identifying key driver mutations, a study evaluating the effect of TP53 mutation VAF on survival in hematological malignancies found that a VAF greater than 6% was associated with profoundly shorter survival and poorer prognosis compared to patients with a TP53 VAF below 6%.^17^ This indicates that mutations with a VAF above 6% are clinically relevant. Although a threshold of 8% is higher than the 6% threshold identified in that study, it was selected to increase the stringency of this analysis, ensuring that only the most clinically impactful genetic alterations are included while excluding irrelevant low-frequency mutations.

While PDOs may closely mimic the original tumor material, studies have shown that the BME hydrogel, used for culturing PDOs, can limit viral spread and penetration which potentially reduces OV-induced cell death and thereby affecting EC_50_ estimates.^36^^,^^37^ However, our previous study evaluating NDV-induced cell death in PDOs cultured in both 40% and 80% BME hydrogel did not find an effect of BME hydrogel concentration on NDV-induced cell death.^38^ It is possible that the impact of BME hydrogel on OV-induced cell death might vary between viruses, which could explain the need for high MOIs of RV Jin-3 to induce 50% cell death in our model. Therefore, additional testing of RV Jin-3 induced cell death in various concentrations of BME hydrogel is needed. Although the BME hydrogel could be considered a limitation of the model, it mimics several components of the extracellular matrix that surrounds PDAC tumors in patients.^39^

This study evaluated PDOs only without immune or stromal components; thus, our readouts capture PDO-intrinsic oncolysis rather than immune-mediated effects. Future studies should incorporate autologous or donor peripheral blood mononuclear cells and cancer associated fibroblasts in co-culture to assess OV-driven immune activation and cytotoxicity. However, direct co-culture requires media compromises across cell types that can alter composition or viability.^40^ These experiments are outside the scope of this study.

Identifying relevant genetic markers by correlating intracellular abnormalities with sensitivity to OV therapy through transcriptome analysis remains a substantial challenge. Transcriptomic analyses of PDOs provide only a snapshot of their expression profile, which may overlook potential genetic biomarkers. This limitation is further fortified by variations in RNA isolation timing, various phases of cell proliferation cycles, differing passage numbers, and varying confluency levels for each PDO. To address this and improve the robustness and completeness of expression profiles in future studies, transcriptomes should ideally be generated at various passage levels and time points after passaging.

Neoadjuvant chemotherapy has been shown to potentially cause histological and morphological changes^41^^,^^42^ and induce drug resistance in several cancers, including PDAC, which could affect the response to OV therapy and the identification of associated GEPs.^43^ Here, histological analysis of the primary tumor material from patient 1 (treatment-naive) and patient 5 (FOLFIRINOX-treated) revealed profound differences in nucleus density. Although this suggests that pre-treatment with FOLFIRINOX may affect tumor histology, these variations could also be attributed to other tumor characteristics such as disease stage. For instance, the tumor of patient 1 was classified as stage IB, while tumor of patient 5 was stage IIB. Conversely, analyses of the PDOs from these patients showed no major histological differences, which suggests that FOLFIRINOX treatment did not impact the histology of PDOs. Similarly, in other studies with FOLFIRINOX-treated PDAC tumors, original tumor material revealed histological differences, whereas the corresponding PDOs did not exhibit any differences.^9^ Analysis of the morphology between PDO-6 (treatment-naive) and PDO-7 (FOLFIRINOX-treated) using brightfield microscopy demonstrated that FOLFIRINOX treatment possibly did not have impact on the morphology of PDAC PDOs, as both exhibited a similarly dense morphology. Our findings revealed that FOLFIRINOX treatment did not influence the sensitivity of PDOs to OV therapy, as PDO-5 was sensitive to NDV, while PDO-10 was not, despite both being treated with FOLFIRINOX. Altogether, the results of our sample set of 10 PDOs suggest that FOLFIRINOX treatment possibly did not affect the histology, morphology or sensitivity to OV therapy in PDAC PDOs.

In this study, we identified GEPs from PDAC PDOs with potential predictive value for OV therapy. Given the complexity of the interactions of OVs with multiple cancer cell pathways, it is unlikely that a single gene or pathway will solely determine sensitivity to OV therapy. While our findings provide a foundation for identifying GEPs as potential predictive markers of OV sensitivity, future studies should include larger cohorts of PDAC PDOs to increase the robustness and reliability of the correlation between transcriptomes and sensitivity to OV therapy. Multiple, similar, studies could identify overlapping genes in GEPs across these studies, which can further refine pools of candidate genes with predictive value for sensitivity to OV therapy. Subsequently, the most consistently identified genes in GEPs should be validated in a clinical setting. Ultimately, validated genes with predictive value for OV therapy could pave the way for a personalized medicine approach, allowing OV therapy to be tailored to individual PDAC patients.

## Materials and methods

### Patient-derived samples

Ten fresh tumor samples were collected from PDAC patients that were part of a clinical study described previously.^9^ These ten PDAC tumor samples were selected to ensure coverage of diverse tumor grades (IA to III), prior therapy status (naive vs. neoadjuvant), and differentiation (moderate to poor). This sample size was deemed sufficient for proof-of-principle analyses, yet feasible given the limited availability of patient tumor material. This study was conducted following the principles of the Declaration of Helsinki and approved by the local medical ethics committee (MEC-2015-085). All patients provided written informed consent. Tumor material was retrieved from both the experimental and treatment naive arms of the study.

### Establishment and culturing of patient-derived organoids

Resected human PDAC tumor tissues were processed and PDOs were established and cultured as described before using 80% Cultrex Reduced Growth Factor Basement Membrane Extract, Type R1 (BME hydrogel; Bio-Techne, no. 3433-010-R1) and 20% AdDMEM/F12 (Gibco, no. 12634028) supplemented with 1× Glutamax (Gibco, no. 35050061), 1× HEPES (Gibco, no. 15630), and 100 μg/mL Primocin (InvivoGen, no. ant-pm-1).^9^^,^^11^ PDOs between passage levels 5 and 15 were cultured until ready for expansion or inoculation with OVs.

### Oncolytic virus production

Virus stocks of RV Jin-3 and Goravir (kind gift of Rob C. Hoeben) were produced and titrated in HER911 cells,^44^ while those of MeV-Edm were produced and titrated in Vero cells.^15^^,^^45^ Virus stock of lentogenic NDV LaSota strain (NDV) was produced in embryonated chicken eggs, as described previously.^46^ All virus stocks of NDV were titrated in Vero cells (RRID:CVCL_0059). Infectious titers were calculated using the method of Reed & Muench and expressed as median tissue culture infectious dose (TCID_50_/mL).^47^

### Determining sensitivity of PDOs to a panel of OVs

Each single well of a 24-well plate consisted of one BME hydrogel dome containing multiple PDOs, where the number and size of the PDOs varied per dome. To prepare for inoculation, PDOs were extracted and pooled from four BME hydrogel domes using Cell Recovery Solution (Corning, no. 354253). The PDOs were washed with basic media, mechanically fragmented in the same medium, and reseeded into 12 new BME hydrogel domes at a 1:3 dilution ratio, with each well containing 40 μL of BME hydrogel and 10 μL of basic media. Once the PDOs covered most of the culture area within the dome, roughly 80% confluency, PDOs from one dome were used to count cells. To this end, PDOs were extracted from the hydrogel domes and digested with TrypLE (Gibco, no. 12604-013) to obtain single cells. Subsequently, the cells were counted using Kova Glastic Slides (Instruchemie, no. 87144) and the determined cell count was used to estimate the number of cells present in the remaining PDOs.

For inoculation, the intact PDOs from the remaining wells were extracted from the domes and evenly distributed into 15 mL Falcon tubes with each tube containing a number of PDOs that totally contained approximately 1 × 10^5^ cells. The intact PDOs were either mock-treated or inoculated at the indicated MOIs in triplicate for one hour at 37°C. Afterward, the intact PDOs were placed back in 80% BME hydrogel and 20% basic media. Inoculated PDOs were cultured for five days after which cell viability was assessed using the CellTiter-Glo 3D Cell Viability assay (Promega, no. G9682) to measure ATP release, following the manufacturer’s instructions. Cell viability is presented as the percentage of luminescence of inoculated cells versus mock inoculated cells, which were considered to have a viability of 100%. All experiments were conducted in triplicates. All half maximal effective concentration (EC_50_) calculations were done using GraphPad Prism 10 (RRID:SCR_002798).

### Histology and immunohistochemistry

PDOs were prepared for histology and immunohistochemistry by fixating domes containing PDOs in 2% paraformaldehyde and 0.2% glutaraldehyde for 2 h at 37°C. Afterward, the fixed domes were mounted with low melting agar and stored at 4°C overnight. Paraffin-embedded blocks were cut into 4-μm sections and mounted on Superfrost plus slides (Menzel-Gläser). For histology analysis, sections were stained with hematoxylin and eosin (H&E; HE600, Ventana, no. 06917259001) and evaluated by a clinical pathologist. IHC staining of the sections was performed for keratin-7 (CK-7) using 0.11 mg/mL concentration of the CK7 mouse monoclonal antibody (clone: A53-B/A2.26; Cell Marque).^48^ Staining was done by automated IHC using the Ventana Benchmark ULTRA (Ventana Medical Systems Inc.).

### DNA/RNA isolation

PDOs were extracted from the domes using Cell Recovery Solution (Corning, no. 354253). DNA and RNA from PDOs were isolated using the AllPrep DNA/RNA Mini Kit (QIAGEN) and concentrations were determined using Nanodrop One C (Thermo Fisher Scientific, no. ND-ONEC-W).

To extract DNA from original patient tumor tissues, samples were formalin-fixed and paraffin-embedded (FFPE). Subsequently, five 10-μm-thick tissue sections from the FFPE blocks were deparaffinized and stained with hematoxylin to identify and delineate tumor areas. To prepare for DNA extraction, delineated tumor areas of fixed tissues were manually microdissected into 5% Chelex 100 Resin (Bio-Rad) and cell lysis solution (Promega) using sterile scalpels. DNA extraction was performed by digesting the lysate with proteinase K (Roche) during an overnight incubation at 56°C. Following digestion, proteinase K was inactivated by heating the sample for 10 min at 95°C. The resulting cell debris was pelleted by centrifugation at 13,300 rpm for 10 min. DNA concentrations in the supernatant were subsequently measured using the Quant-iT Picogreen assay kit (Thermo Fisher Scientific, Grand Island, NY, USA), according to the manufacturer’s instructions.

### Targeted next-generation sequencing

Sequencing of DNA extracted from patient-derived primary tumor tissues and PDOs was performed using a next-generation sequencing (NGS) panel targeting 58 cancer-associated genes including key driver genes, such as *KRAS*, *TP53*, *SMAD4*, and *CDKN2A*, that are frequently mutated in pancreatic ductal adenocarcinoma (PDAC) tumors (Table S1).^49^ For library preparation, the Ion AmpliSeq library kit Plus (Thermo Fisher Scientific) was used to perform two multiplex PCR reactions using 10 ng of DNA per reaction according to manufacturer’s instructions. Libraries were sequenced on the Ion S5XL Semiconductor Sequencer (Thermo Fisher Scientific). To identify relevant cancer-associated mutations, variants were detected and analyzed by VARIANT CALLER v.5.10.0.18 (Thermo Fisher Scientific) and mutations with a variant allele frequency (VAF) of at least 8% were selected.

### RNA sequencing and generation of gene expression profiles

A library of the RNA extracted from PDOs was prepared using the NEBNext Ultra II Directional RNA Library Prep Kit for Illumina (NEB, no. E7760S/L). Total RNA was depleted from ribosomal RNA (rRNA) using the QIAGEN fast select kit (no. 334387). Afterward, cDNA was synthesized and used for ligation to the sequencing adapters and PCR amplification of the resulting product. Sequencing was carried out by GenomeScan (Leiden, the Netherlands) using the NovaSeq6000, following the manufacturer’s protocols with 0.8 nM of cDNA and utilizing NovaSeq control software NCS v.1.8. Sequences from the obtained FastQ files were trimmed to remove low-quality bases and adapter sequences, aligned to the reference genome to map the reads to their corresponding genomic locations, and then counted and normalized using the nf-core pipeline, resulting in the number of transcripts per million reads (TPM).^50^ Long non-coding RNAs, irrelevant genes, including uncharacterized genes (LOCXXX), and genes with expression counts lower than four were removed from all sample sets.

To correlate gene expression profiles (GEPs) with sensitivity of PDOs to OVs, spearman correlation was performed using R (https://www.r-project.org/) on transcript per million (TPM)-normalized gene expression and the EC_50_ values of each OV. Genes with a *p* value <0.05 were considered significant and were not adjusted for multiple comparisons. A negative Spearman correlation coefficient (rho; ρ) indicated that the significant genes were associated with sensitivity to OV therapy, while a positive correlation suggested that these genes were linked to resistance to OV therapy.

Gene Ontology enrichment analysis was performed on GEPs associated with sensitivity and resistance to each OV. GEPs were analyzed using DAVID (Database for Annotation, Visualization, and Integrated Discovery), a functional annotation tool developed by the NIH, which has the Gene Ontology database integrated.^51^^,^^52^ Biological processes in the Gene Ontology database, often referred to as “terms,” were considered significant if their *p* value was less than 0.05.

## Data and code availability

RNA-seq data supporting this study’s findings are available in the Gene Expression Omnibus (GEO: GSE288907). Gene expression profiles generated by RNA-seq and the list of genes analyzed by next-generation sequencing are available as supplementary data. Additional data and materials can be made available upon request.

## Acknowledgments

We would like to acknowledge the support from the Dutch Foundation OAK (Overleven met Alvleesklier kanker) from the Netherlands, grants no. 16.01 and no. 19.10, and Netherlands NWO-TTW grant no. 15414 (NWO-domein Toegepaste en Technische Wetenschappen).

## Author contributions

Conceptualization, M.H., R.A.M.F., and B.G.v.d.H.; data curation, M.H., E.A.F., and F.M.; formal analysis, M.H., J.J., A.S., and B.G.v.d.H.; investigation, M.H., E.A.F., F.M., and J.J.; methodology, M.H., E.A.F., F.M., and J.J.; project administration, M.H., R.A.M.F., and B.G.v.d.H.; visualization, M.H.; supervision, A.S., R.A.M.F., and B.G.v.d.H.; writing – original draft, M.H.; writing –review & editing, R.A.M.F. and B.G.v.d.H.

## Declaration of interests

The authors declare no competing interests.

## Supplemental information

Table S1. Cancer signature gene panel

Table S2. Gene expression profile associated with sensitivity or resistance per OV

## References

[1] International Agency for Research on Cancer (2020).

[2] Sarantis P., Koustas E., Papadimitropoulou A., Papavassiliou A.G., Karamouzis M.V. (2020). Pancreatic ductal adenocarcinoma: Treatment hurdles, tumor microenvironment and immunotherapy. World J. Gastrointest. Oncol..

[3] Buijs P., van Nieuwkoop S., Vaes V., Fouchier R., van Eijck C., van den Hoogen B. (2015). Recombinant immunomodulating lentogenic or mesogenic oncolytic Newcastle Disease Virus for treatment of pancreatic adenocarcinoma. Viruses.

[4] Awano M., Fujiyuki T., Shoji K., Amagai Y., Murakami Y., Furukawa Y., Sato H., Yoneda M., Kai C. (2016). Measles virus selectively blind to signaling lymphocyte activity molecule has oncolytic efficacy against nectin-4-expressing pancreatic cancer cells. Cancer Sci..

[5] Van De Merbel A.F., Van Der Horst G., Van Der Mark M.H., Bots S.T.F., Van Den Wollenberg D.J.M., De Ridder C.M.A., Stuurman D., Aalders T., Erkens-Schulz S., Van Montfoort N. (2022). Reovirus mutant jin-3 exhibits lytic and immune-stimulatory effects in preclinical human prostate cancer models. Cancer Gene Ther..

[6] Lamfers M.L.M., Grill J., Dirven C.M.F., van Beusechem V.W., Geoerger B., Van Den Berg J., Alemany R., Fueyo J., Curiel D.T., Vassal G. (2002). Potential of the Conditionally Replicative Adenovirus Ad5-D24RGD in the Treatment of Malignant Gliomas and Its Enhanced Effect with Radiotherapy. Cancer Res..

[7] Lawler S.E., Speranza M.C., Cho C.F., Chiocca E.A. (2017). Oncolytic Viruses in Cancer Treatment: A Review. JAMA Oncol..

[8] Driehuis E., Kretzschmar K., Clevers H. (2020). Establishment of patient-derived cancer organoids for drug-screening applications. Nat. Protoc..

[9] Farshadi E.A., Chang J., Sampadi B., Doukas M., Van’t Land F., van der Sijde F., Vietsch E.E., Pothof J., Koerkamp B.G., van Eijck C.H.J. (2021). Organoids derived from neoadjuvant FOLFIRINOX patients recapitulate therapy resistance in pancreatic ductal adenocarcinoma. Clin. Cancer Res..

[10] Vlachogiannis G., Hedayat S., Vatsiou A., Jamin Y., Fernández-Mateos J., Khan K., Lampis A., Eason K., Huntingford I., Burke R. (2018). Patient-derived organoids model treatment response of metastatic gastrointestinal cancers. Science.

[11] Driehuis E., Gracanin A., Vries R.G.J., Clevers H., Boj S.F. (2020). Establishment of Pancreatic Organoids from Normal Tissue and Tumors. STAR Protoc..

[12] Corrò C., Novellasdemunt L., Li V.S.W. (2020). A brief history of organoids. Am. J. Physiol. Cell Physiol..

[13] Li Y., Tang P., Cai S., Peng J., Hua G. (2020). Organoid based personalized medicine: from bench to bedside. Cell Regen..

[14] Domingo-Musibay E., Allen C., Kurokawa C., Hardcastle J.J., Aderca I., Msaouel P., Bansal A., Jiang H., DeGrado T.R., Galanis E. (2014). Measles Edmonston vaccine strain derivatives have potent oncolytic activity against osteosarcoma. Gene Ther..

[15] Bots S.T.F., Landman S.L., Rabelink M.J.W.E., van den Wollenberg D.J.M., Hoeben R.C. (2023). Immunostimulatory Profile of Cancer Cell Death by the AdV-Lumc007-Derived Oncolytic Virus ‘GoraVir’ in Cultured Pancreatic Cancer Cells. Viruses.

[16] Conroy T., Hammel P., Hebbar M., Ben Abdelghani M., Wei A.C., Raoul J.L., Choné L., Francois E., Artru P., Biagi J.J. (2018). FOLFIRINOX or Gemcitabine as Adjuvant Therapy for Pancreatic Cancer. N. Engl. J. Med..

[17] Belickova M., Vesela J., Jonasova A., Pejsova B., Votavova H., Merkerova M.D., Zemanova Z., Brezinova J., Mikulenkova D., Lauermannova M. (2016). *TP53* mutation variant allele frequency is a potential predictor for clinical outcome of patients with lower-risk myelodysplastic syndromes. Oncotarget.

[18] Kasuya H., Nishiyama Y., Nomoto S., Goshima F., Takeda S., Watanabe I., Nomura N., Shikano T., Fujii T., Kanazumi N., Nakao A. (2007). Suitability of a US3-inactivated HSV mutant (L1BR1) as an oncolytic virus for pancreatic cancer therapy. Cancer Gene Ther..

[19] Buijs P.R.A., Van Eijck C.H.J., Hofland L.J., Fouchier R.A.M., Van Den Hoogen B.G. (2014). Different responses of human pancreatic adenocarcinoma cell lines to oncolytic Newcastle disease virus infection. Cancer Gene Ther..

[20] Raimondi G., Mato-Berciano A., Pascual-Sabater S., Rovira-Rigau M., Cuatrecasas M., Fondevila C., Sánchez-Cabús S., Begthel H., Boj S.F., Clevers H., Fillat C. (2020). Patient-derived pancreatic tumour organoids identify therapeutic responses to oncolytic adenoviruses. EBioMedicine.

[21] Lin D., Shen Y., Liang T. (2023). Oncolytic virotherapy: basic principles, recent advances and future directions. Sig. Transduct. Target Ther.

[22] Boj S.F., Hwang C.I., Baker L.A., Chio I.I.C., Engle D.D., Corbo V., Jager M., Ponz-Sarvise M., Tiriac H., Spector M.S. (2015). Organoid models of human and mouse ductal pancreatic cancer. Cell.

[23] Raimondi G., Mato-Berciano A., Pascual-Sabater S., Rovira-Rigau M., Cuatrecasas M., Fondevila C., Sánchez-Cabús S., Begthel H., Boj S.F., Clevers H., Fillat C. (2020). Patient-derived pancreatic tumour organoids identify therapeutic responses to oncolytic adenoviruses. EBioMedicine.

[24] Vazaios K., Stavrakaki Ε., Vogelezang L.B., Ju J., Waranecki P., Metselaar D.S., Meel M.H., Kemp V., Van Den Hoogen B.G., Hoeben R.C. (2024). The heterogeneous sensitivity of pediatric brain tumors to different oncolytic viruses is predicted by unique gene expression profiles. Mol. Ther. Oncol..

[25] Schäfer T.E., Knol L.I., Haas F.V., Hartley A., Pernickel S.C.S., Jády A., Finkbeiner M.S.C., Achberger J., Arelaki S., Modic Ž. (2024). Biomarker screen for efficacy of oncolytic virotherapy in patient-derived pancreatic cancer cultures. EBioMedicine.

[26] Kim Y., Kim I., Shin K. (2023). A new era of stem cell and developmental biology: from blastoids to synthetic embryos and beyond. Exp. Mol. Med..

[27] Van Den Hengel S.K., Balvers R.K., Dautzenberg I.J.C., Van Den Wollenberg D.J.M., Kloezeman J.J., Lamfers M.L., Sillivis-Smit P.A.E., Hoeben R.C. (2013). Heterogeneous reovirus susceptibility in human glioblastoma stem-like cell cultures. Cancer Gene Ther..

[28] Marcato P., Dean C.A., Giacomantonio C.A., Lee P.W.K. (2009). Oncolytic Reovirus Effectively Targets Breast Cancer Stem Cells. Mol. Ther..

[29] Kazimirsky G., Jiang W., Slavin S., Ziv-Av A., Brodie C. (2016). Mesenchymal stem cells enhance the oncolytic effect of Newcastle disease virus in glioma cells and glioma stem cells via the secretion of TRAIL. Stem Cell Res. Ther..

[30] Coffey M.C., Strong J.E., Forsyth P.A., Lee P.W. (1998). Reovirus Therapy of Tumors with Activated Ras Pathway. Science.

[31] Korzeniecki C., Priefer R. (2021). Targeting KRAS mutant cancers by preventing signaling transduction in the MAPK pathway. Eur. J. Med. Chem..

[32] Stepanenko A.A., Sosnovtseva A.O., Valikhov M.P., Chernysheva A.A., Cherepanov S.A., Yusubalieva G.M., Ruzsics Z., Lipatova A.V., Chekhonin V.P. (2022). Superior infectivity of the fiber chimeric oncolytic adenoviruses Ad5/35 and Ad5/3 over Ad5-delta-24-RGD in primary glioma cultures. Mol. Ther. Oncol..

[33] Fueyo J., Alemany R., Gomez-Manzano C., Fuller G.N., Khan A., Conrad C.A., Liu T.J., Jiang H., Lemoine M.G., Suzuki K. (2003). Preclinical Characterization of the Antiglioma Activity of a Tropism-Enhanced Adenovirus Targeted to the Retinoblastoma Pathway. J. Natl. Cancer Inst..

[34] Wang L., Liu W., Li Z., Wang X., Feng X., Wang Z., Wu J., Zhang H., Wu H., Kong W. (2020). A tropism-transformed Oncolytic Adenovirus with Dual Capsid Modifications for enhanced Glioblastoma Therapy. J. Cancer.

[35] Frappart P.O., Walter K., Gout J., Beutel A.K., Morawe M., Arnold F., Breunig M., Barth T.F., Marienfeld R., Schulte L. (2020). Pancreatic cancer-derived organoids – a disease modeling tool to predict drug response. United Eur Gastroenterol J.

[36] Wadstrom T., Ljungh Å. (1999). Glycosaminoglycan-binding microbial proteins in tissue adhesion and invasion: key events in microbial pathogenicity. J. Med. Microbiol..

[37] Kloker L.D., Yurttas C., Lauer U.M. (2018). Three-dimensional tumor cell cultures employed in virotherapy research. Oncolytic Virother..

[38] Huberts M., Farshadi E.A., Groeneveld D., Fouchier R.A.M., Van Den Hoogen B.G. (2025). The use of pancreatic ductal adenocarcinoma 2D and 3D models to evaluate NDV infection, replication and induced cell death. Sci. Rep..

[39] Henke E., Nandigama R., Ergün S. (2020). Extracellular Matrix in the Tumor Microenvironment and Its Impact on Cancer Therapy. Front. Mol. Biosci..

[40] Tsai S., McOlash L., Palen K., Johnson B., Duris C., Yang Q., Dwinell M.B., Hunt B., Evans D.B., Gershan J., James M.A. (2018). Development of primary human pancreatic cancer organoids, matched stromal and immune cells and 3D tumor microenvironment models. BMC Cancer.

[41] Honkoop A.H., Pinedo H.M., De Jong J.S., Verheul H.M., Linn S.C., Hoekman K., Wagstaff J., Van Diest P.J. (1997). Effects of Chemotherapy on Pathologic and Biologic Characteristics of Locally Advanced Breast Cancer. Am. J. Clin. Pathol..

[42] Jaime Dos Santos B., Balabram D., Mara Reis Gomes V., Costa Café De Castro C., Henrique Costa Diniz P., Araújo Buzelin M., Buzelin Nunes C. (2024). Changes in Invasive Breast Carcinomas after Neoadjuvant Chemotherapy Can Influence Adjuvant Therapeutic Decisions. Cancer Res. Treat..

[43] Quiñonero F., Mesas C., Doello K., Cabeza L., Perazzoli G., Jimenez-Luna C., Rama A.R., Melguizo C., Prados J. (2019). The challenge of drug resistance in pancreatic ductal adenocarcinoma: a current overview. Cancer Biol. Med..

[44] Fallaux F.J., Kranenburg O., Cramer S.J., Houweling A., Van Ormondt H., Hoeben R.C., Van Der Eb A.J. (1996). Characterization of 911: A New Helper Cell Line for the Titration and Propagation of Early Region 1-Deleted Adenoviral Vectors. Hum. Gene Ther..

[45] Dautzenberg I.J.C., Van Den Wollenberg D.J.M., Van Den Hengel S.K., Limpens R.W.A., Bárcena M., Koster A.J., Hoeben R.C. (2014). Mammalian orthoreovirus T3D infects U-118 MG cell spheroids independent of junction adhesion molecule-A. Gene Ther..

[46] Huberts M., De Graaf J.F., Groeneveld D., Van Nieuwkoop S., Fouchier R.A.M., Van Den Hoogen B.G. (2025). Cell-derived Newcastle disease virus variant with two amino acid substitutions near cleavage site of F shows favorable traits as oncolytic virus. Mol. Ther. Oncol..

[47] Reed L.J., Muench H. (1938). A Simple Method of Estimating Fifty Per Cent Endpoints. Am. J. Hyg..

[48] Yoon S., Li H., Quintanar L., Armstrong B., Rossi J.J. (2020). Uncovering Differently Expressed Markers and Heterogeneity on Human Pancreatic Cancer. Transl. Oncol..

[49] Feng H.H., Ye Z., Qin Y., Wu X.X., Jun Y.X., Feng Z.Q., Rong J.S. (2021). Mutations in key driver genes of pancreatic cancer: molecularly targeted therapies and other clinical implications. Acta Pharmacol. Sin..

[50] Ewels P.A., Peltzer A., Fillinger S., Patel H., Alneberg J., Wilm A., Garcia M.U., Di Tommaso P., Nahnsen S. (2020). The nf-core framework for community-curated bioinformatics pipelines. Nat. Biotechnol..

[51] Huang D.W., Sherman B.T., Lempicki R.A. (2009). Systematic and integrative analysis of large gene lists using DAVID bioinformatics resources. Nat. Protoc..

[52] Sherman B.T., Hao M., Qiu J., Jiao X., Baseler M.W., Lane H.C., Imamichi T., Chang W. (2022). DAVID: a web server for functional enrichment analysis and functional annotation of gene lists (2021 update). Nucleic Acids Res..

